# Supplementary figures and images for: Hantavirus infection-induced B cell activation elevates free light chains levels in circulation
Source: PLoS Pathog. 2021 Aug 11;17(8):e1009843. doi: 10.1371/journal.ppat.1009843 (PMC8382192; doi:10.1371/journal.ppat.1009843)

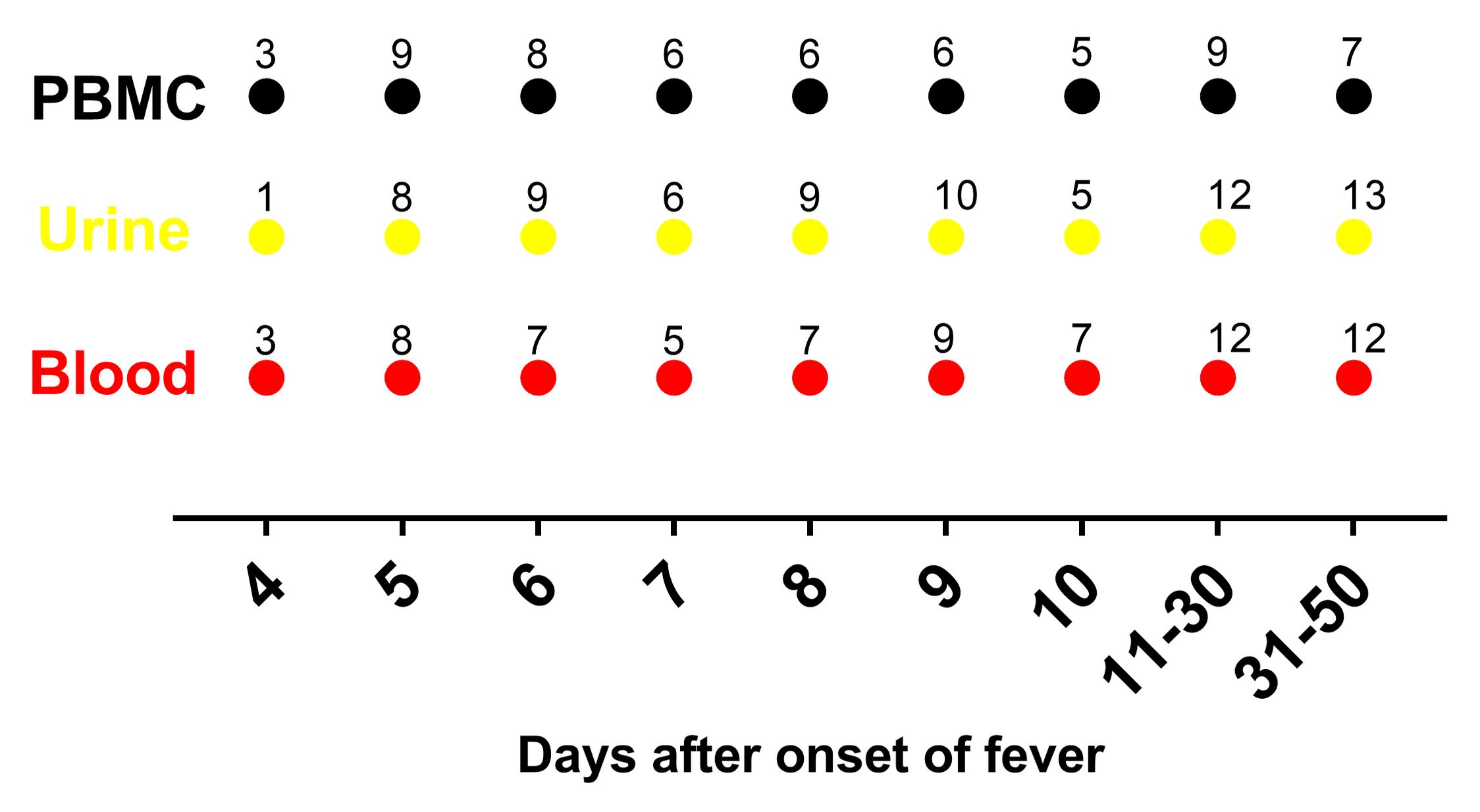

Supplement: S1 Fig — The number of samples at each day post onset of fever are indicated for PBMC, serum and urine obtained from PUUV-caused HFRS patients. (TIF) [file ppat.1009843.s001.tif]

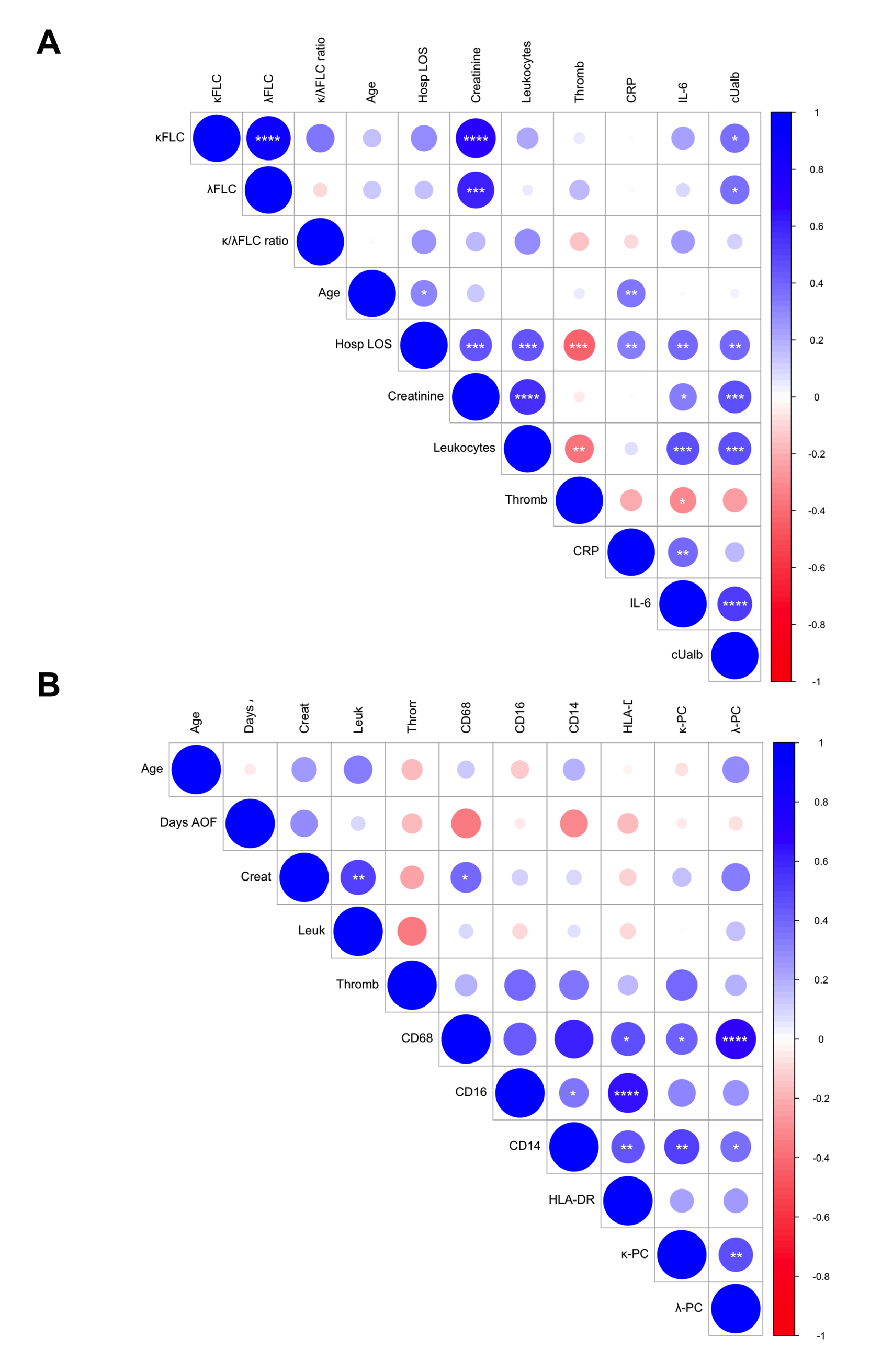

Supplement: S2 Fig — Spearman correlation analysis of serum κFLC, λFLCs and their ratio (in A) and κ- and λ-LC specific plasma cells (PC) in kidneys (in B) with clinical and laboratory parameters are shown. The color of the circles indicates the value of Spearman correlation coefficients as depicted in the legend on the right and increasing size of the circles indicate decreasing p-value (statistical significance is reported as * = p < 0.05, ** = p < 0.01, *** = p < 0.001 and **** = p < 0.0001). Creatinine, leukocytes, C-reactive protein (CRP), interleukin (IL)-6 and thrombocytes correspond to maximum and minimum values in blood during hospital stay, respectively. Hosp. LOS = Length of stay in hospital. cUAlb = Overnight urinary albumin excretion. CD68, CD14, CD16 and HLA-DR correspond to the extent infiltration of CD68+, CD14+, CD16+ and HLA-DR+ leukocytes in kidneys. (TIF) [file ppat.1009843.s002.tif]

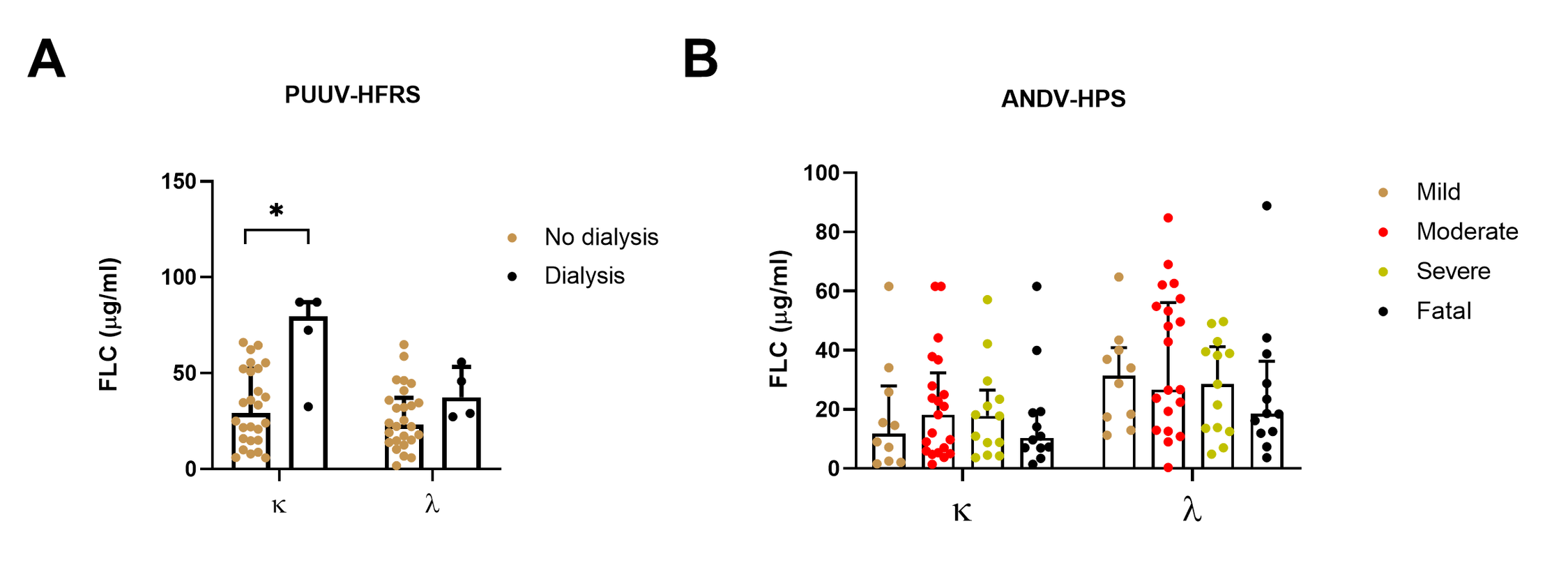

Supplement: S3 Fig — (A) Serum κ and λ FLC levels in PUUV-caused HFRS patients with and without the need of dialysis (n = 26 and n = 4) and (B) ANDV-caused HPS patients stratified based on disease severity (1 = with prodromal symptoms without respiratory involvement; 2 = mild to moderate respiratory compromise without hemodynamic compromise; 3 = with severe respiratory insufficiency with hemodynamic compromise; 4 = with severe respiratory insufficiency with refractory-to-treatment hemodynamic compromise, with a final fatal outcome). Statistically significant differences assessed with Mann-Whitney test and reported as * = p < 0.05. The bars indicate medians + interquartile ranges. (TIF) [file ppat.1009843.s003.tif]

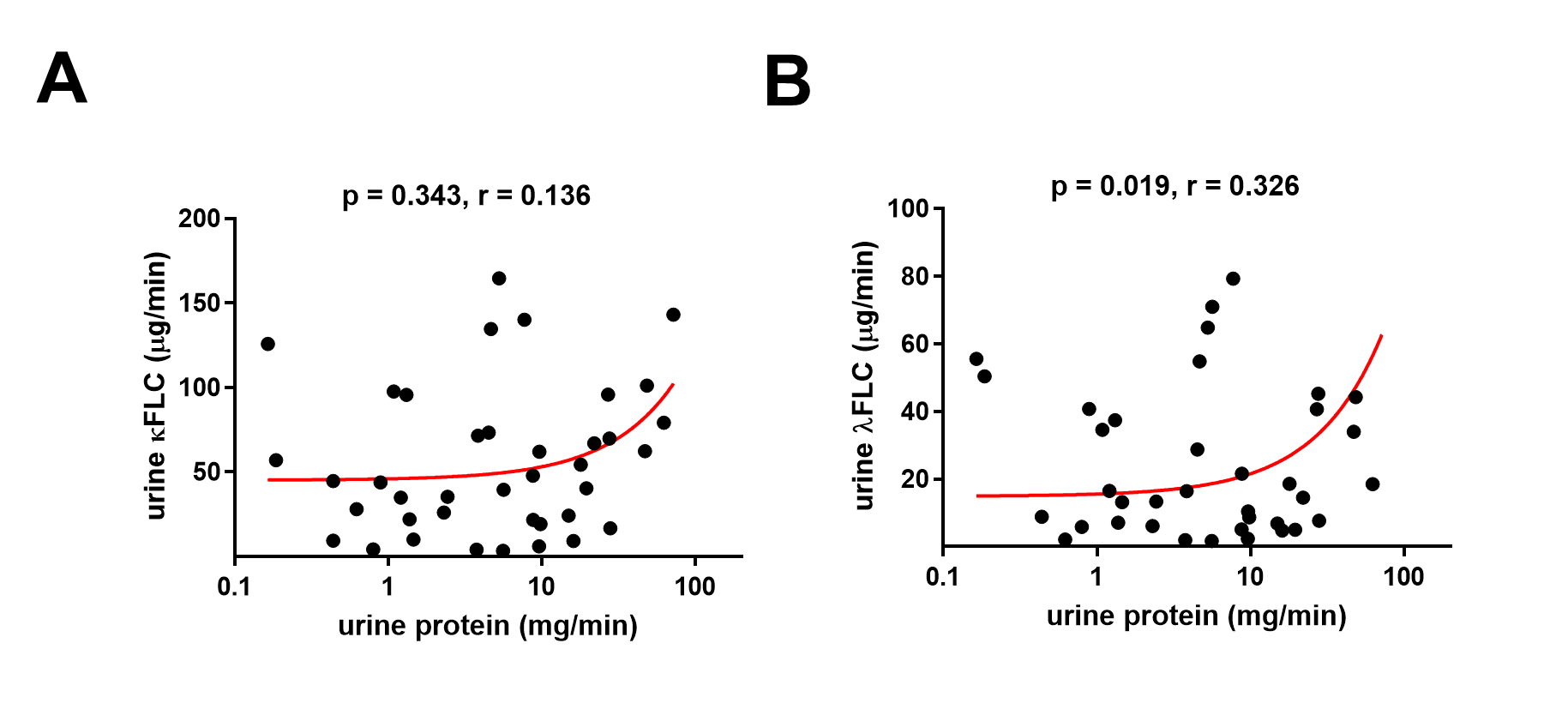

Supplement: S4 Fig — The concentration of FLCs (κ in A and λ in B) in urine samples collected at indicated days post onset of fever from patients with acute PUUV-HFRS (n = 13) were correlated to total urinary protein levels using Spearman’s rank correlation coefficient. Non-linear association between parameters is depicted by the red line. (TIF) [file ppat.1009843.s004.tif]

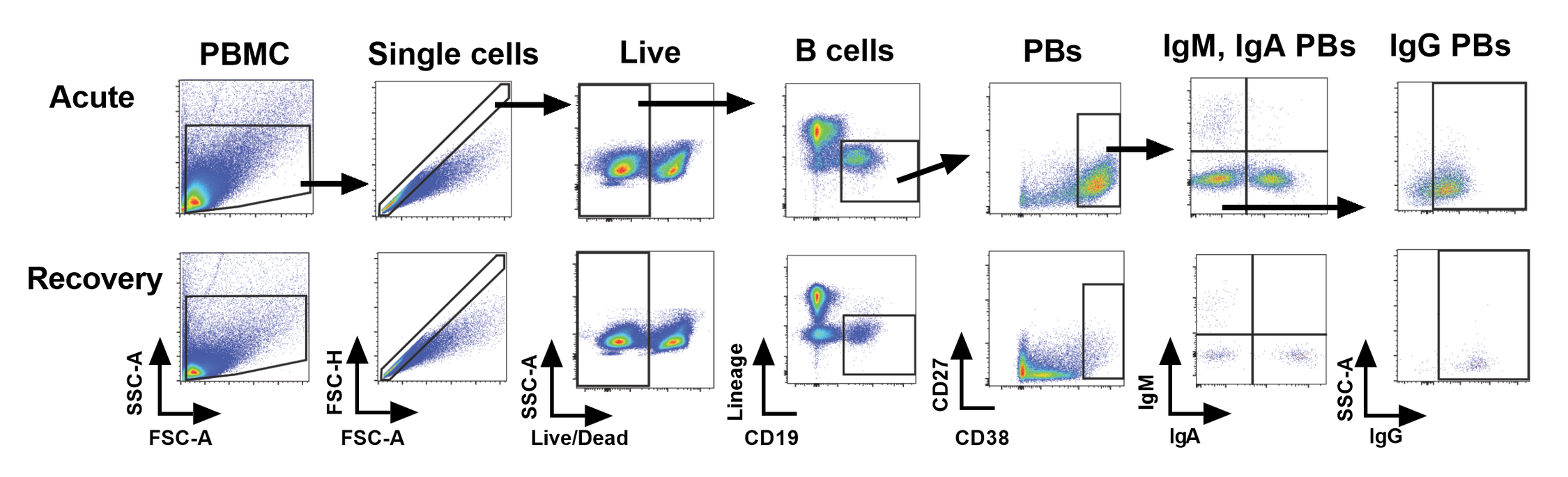

Supplement: S5 Fig — After gating on PBMCs (SSC-A vs. FSC-A), single cells (FSC-H vs. FSC-A) and CD19+ B cells (CD3, CD14, CD56, CD66 vs. CD19), were identified as CD27+CD38++ cells (CD27 vs. CD38). IgM+ and IgA+ PBs were gated from the total PB fraction (IgM vs. IgA) and IgG+ PBs (SSC-A vs. IgG) from the IgM-IgA- fraction. Representative plots for an acute and recovery stage PUUV-HFRS are shown. (TIF) [file ppat.1009843.s005.tif]

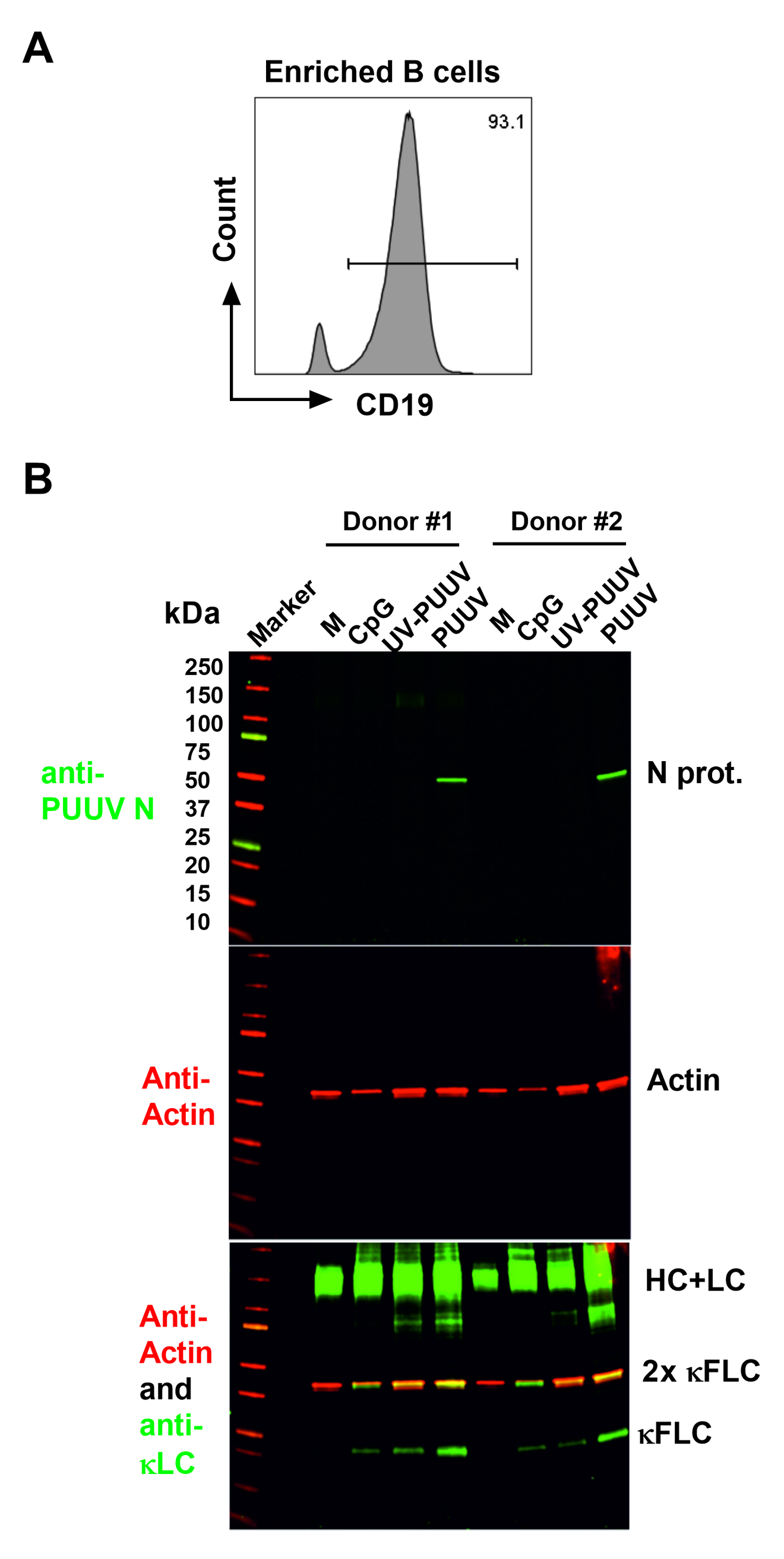

Supplement: S6 Fig — B cells were isolated from healthy volunteer PBMCs by negative selection and a representative histogram of the percentage of CD19+ B cells in the isolated fraction as assessed by flow cytometry is shown in (A). (B) Isolated B cells were left untreated, activated with CpG (1 μM) or infected with live (5 FFFU / cell) or UV-inactivated PUUV and subjected to non-reducing western blot at 5 days post infection. The viral N protein and κLC expressions (both green) were detected by specific Abs produced in mouse or goat, respectively. A rabbit Ab to actin (red) served as a protein loading control. Free and heavy chain (HC)-associated light chains (LC) are indicated. (TIF) [file ppat.1009843.s006.tif]
