# Supplementary material for: Hantavirus infection-induced B cell activation elevates free light chains levels in circulation
Source: PLoS Pathog. 2021 Aug 11;17(8):e1009843. doi: 10.1371/journal.ppat.1009843 (PMC8382192; doi:10.1371/journal.ppat.1009843)
Supplement: S1 Table — (DOCX) [file ppat.1009843.s007.docx]

S1 Table. Clinical and laboratory parameters of HFRS patients with blood samples

| ID | Sex | Age | Creatinine max (µmol/L) | Days after onset | *serum κFLC (µg/ml) | *serum λFLC (μg/ml) | serum κFLCmax (μg/ml) | serum λFLCmax (μg/ml) | urine κFLCmax (μg/L) | urine λFLCmax (μg/L) | PBmax (% of PBMC) |
| --- | --- | --- | --- | --- | --- | --- | --- | --- | --- | --- | --- |
|  |  |  |  |  |  |  |  |  |  |  |  |
| HFRS-1 | F | 34 | 75,0 | 3 | 10,0 | 12,5 | 194,3 | 85,0 | 17,9 | 19,3 | 0,7 |
| HFRS-2 | M | 35 | 113,0 | 5 | 33,4 | 34,6 | 95,4 | 88,6 | 33,4 | 34,6 | 3,0 |
| HFRS-3 | M | 38 | 219,0 | 4 | 25,1 | 17,4 | 190,8 | 163,1 | 43,5 | 39,9 | 1,5 |
| HFRS-4 | F | 49 | 950,0 | 4 | 64,5 | 41,0 | 357,1 | 102,0 | 75,2 | 70,8 | 1,6 |
| HFRS-5 | M | 54 | 140,0 | 6 | 24,1 | 22,2 | 78,8 | 8,5 | 24,1 | 22,2 | 0,4 |
| HFRS-6 | M | 60 | 282,0 | 1 | 21,7 | 14,0 | 411,6 | 133,9 | 39,9 | 32,4 | 0,8 |
| HFRS-7 | M | 31 | 256,0 | 4 | 22,1 | 31,8 | 33,7 | 60,6 | 25,0 | 34,0 | 0,1 |
| HFRS-8 | M | 38 | 369,0 | 3 | 6,1 | 10,3 | 244,1 | 133,3 | 20,6 | 27,5 |  |
| HFRS-9 | F | 36 | 200,0 | 3 | 7,9 | 1,8 | 223,7 | 50,2 | 29,1 | 13,1 |  |
| HFRS-10 | M | 46 | 445,0 | 5 | 66,0 | 46,6 |  |  |  |  |  |
| HFRS-11 | M | 58 | 344,0 | 5 | 40,6 | 44,6 |  |  |  |  |  |
| HFRS-12 | M | 43 | 480,0 | 6 | 55,4 | 32,2 |  |  |  |  |  |
| HFRS-13 | M | 52 | 118,0 | 3 | 52,4 | 33,0 | 564,6 | 500,0 | 44,1 | 39,5 |  |
| HFRS-14 | F | 34 | 70,0 | 4 | 14,8 | 6,8 |  |  |  |  | 0,7 |
| HFRS-15 | M | 38 | 772,0 | 3 | 32,5 | 27,3 | 440,7 | 230,7 | 69,8 | 79,1 | 5,1 |
| HFRS-16 | M | 36 | 1285,0 | 3 | 72,4 | 29,0 | 3326,0 | 1745,1 | 80,4 | 74,5 |  |
| HFRS-17 | M | 25 | 906,0 | 5 | 62,3 | 65,0 |  |  |  |  |  |
| HFRS-18 | M | 55 | 92,0 | 4 | 8,8 | 17,9 |  |  |  |  |  |
| HFRS-19 | M | 52 | 1156,0 | 5 | 87,0 | 55,8 |  |  |  |  |  |
| HFRS-20 | F | 52 | 65,0 | 6 | 15,0 | 6,0 | 3,3 | 0,1 | 15,0 | 6,0 |  |
| HFRS-21 | M | 22 | 88,0 | 1 | 34,7 | 25,4 |  |  |  |  |  |
| HFRS-22 | F | 71 | 79,0 | 3 | 20,9 | 22,3 |  |  |  |  |  |
| HFRS-23 | F | 45 | 386,0 | 4 | 37,5 | 19,1 | 65,0 | 64,9 | 59,3 | 47,4 |  |
| HFRS-24 | M | 61 | 143,0 | 2 | 35,9 | 24,1 |  |  |  |  |  |
| HFRS-25 | M | 50 | 917,0 | 6 | 55,5 | 58,8 |  |  |  |  | 0,5 |
| HFRS-26 | F | 55 | 243,0 | 4 | 52,4 | 46,2 |  |  |  |  | 9,3 |
| HFRS-27 | M | 42 | 92,0 | 3 | 5,9 | 15,2 |  |  |  |  |  |
| HFRS-28 | F | 57 | 297,0 | 5 | 50,8 | 35,9 |  |  |  |  |  |
| HFRS-29 | F | 58 | 543,0 | 1 | 87,0 | 45,8 |  |  |  |  |  |
| HFRS-30 | M | 55 | 372,0 | 3 | 15,9 | 14,8 |  |  |  |  | 1,9 |
| HFRS-31 | M | 57 | 97,0 | 4 |  |  |  |  |  |  | 0,3 |

max = maximum values in longitudinal samples obtained during hospitalization

PB = plasmablasts

Days after onset = Days after onset of fever at admission

*= measured at 1^st^ day of hospitalization
