# Supplementary material for: Hantavirus infection-induced B cell activation elevates free light chains levels in circulation
Source: PLoS Pathog. 2021 Aug 11;17(8):e1009843. doi: 10.1371/journal.ppat.1009843 (PMC8382192; doi:10.1371/journal.ppat.1009843)
Supplement: S2 Table — (DOCX) [file ppat.1009843.s008.docx]

S2 Table. Clinical characteristics of HPS patients

| ID | Sex | Age | Severity* | λFLC (ug/ml) | λFLC (ug/ml) | Days after onset | Renal dysfunction** |
| --- | --- | --- | --- | --- | --- | --- | --- |
| HPS-1 | F | 26 | 3 | 8,5 | 13,1 | 8 | NA |
| HPS-2 | M | 32 | 3 | 10,2 | 31,5 | 5 | NA |
| HPS-3 | F | 30 | 3 | 25,4 | 34,3 | 9 | NO |
| HPS-4 | M | 45 | 3 | 47,8 | 39 | 8 | YES |
| HPS-5 | F | 31 | 3 | 35,6 | 31,9 | 8 | YES |
| HPS-6 | M | 38 | 3 | 4,8 | 24,3 | 11 | NO |
| HPS-7 | M | 30 | 2 | 4,4 | 4,7 | 7 | NO |
| HPS-8 | F | 28 | 2 | 32,1 | 38,9 | 7 | NO |
| HPS-10 | M | 32 | 2 | 24 | 47,6 | 6 | NO |
| HPS-11 | M | 33 | 2 | 38,1 | 41,6 | 2 | YES |
| HPS-15 | M | 26 | 2 | 6,1 | 23 | 3 | NO |
| HPS-16 | M | 25 | 1 | 7,1 | 32,3 | 10 | NO |
| HPS-17 | M | 53 | 1 | 8,6 | 12,3 | 3 | NO |
| HPS-18 | F | 34 | 4 | 7,2 | 24,4 | 4 | NO |
| HPS-19 | F | 26 | 4 | 12,8 | 17,4 | 2 | YES |
| HPS-20 | M | 52 | 4 | 16,1 | 31,9 | 6 | YES |
| HPS-21 | M | 21 | 1 | 3 | 13,5 | 4 | NO |
| HPS-22 | M | 39 | 1 | 22,2 | 72,7 | 6 | NO |
| HPS-23 | F | 25 | 2 | 19,9 | 20,3 | 6 | YES |
| HPS-24 | M | 17 | 2 | 7 | 10,7 | 6 | NO |
| HPS-25 | M | 31 | 1 | 3,4 | 30,1 | 5 | NO |
| HPS-26 | M | 68 | 2 | 31,2 | 63,3 | 9 | NO |
| HPS-27 | F | 43 | 3 | 16,1 | 14,1 | 2 | NO |
| HPS-28 | F | 30 | 4 | 2,4 | 15,7 | 3 | NO |
| HPS-29 | M | 65 | 2 | 8,6 | 13,2 | 6 | NO |
| HPS-30 | F | 64 | 4 | 9,2 | 7 | 2 | NO |
| HPS-31 | M | 57 | 3 | 15,7 | 13,9 | 6 | NO |
| HPS-32 | F | 44 | 3 | 5 | 9,3 | 5 | NO |
| HPS-33 | M | 35 | 3 | 20,4 | 19,4 | 2 | NO |
| HPS-34 | F |  | 3 | 4,3 | 38,5 | 3 | NO |
| HPS-35 | F | 24 | 2 | 5,4 | 12 | 2 | NO |
| HPS-36 | F |  | 4 | 7 | 12,8 | 2 | NO |
| HPS-37 | M |  | 4 | 7 | 9,5 | 4 | NO |
| HPS-38 | M |  | 2 | 16,1 | 17,9 | 13 | NO |
| HPS-39 | M | 27 | 3 | 8,4 | 7,8 | 4 | NO |
| HPS-40 | F | 49 | 4 | 4,1 | 17,3 | 2 | YES |
| HPS-41 | M | 42 | 2 | 5,7 | 13,4 | 8 | YES |
| HPS-42 | F | 59 | 1 | 13,1 | 17,2 | 5 | NO |
| HPS-43 | F | 26 | 1 | 29,1 | 24,4 | 5 | NO |
| HPS-44 | M | 39 | NA | 28,3 | 21,9 | 5 | NO |
| HPS-45 | F | 50 | 4 | 10,2 | 13,2 | 7 | YES |
| HPS-46 | M | 32 | 2 | 5,1 | 20,9 | 5 | NO |
| HPS-47 | M | 23 | 2 | 18,4 | 48 | 8 | NO |
| HPS-48 | M | 42 | 4 | 16,6 | 35,1 | 7 | YES |
| HPS-50 | M | 24 | 2 | 11,1 | 22,8 | 6 | NO |
| HPS-52 | F | 23 | 2 | 9,3 | 44,3 | 6 | YES |
| HPS-53 | M | 29 | 1 | 13,9 | 28,1 | 5 | NO |
| HPS-54 | M | 40 | 2 | 20,6 | 34,2 | 6 | NO |
| HPS-56 | M | 30 | 3 | 18,5 | 31,1 | 11 | YES |
| HPS-57 | M | 49 | 2 | 21,7 | 42,5 | 4 | NO |
| HEALTHY-1 | F |  |  | 2,7 | 6,3 |  | NO |
| HEALTHY-2 | F |  |  | 4,9 | 4,2 |  | NO |
| HEALTHY-3 | F |  |  | 12 | 11,4 |  | NO |
| HEALTHY-4 | M |  |  | 2,9 | 7 |  | NO |
| HEALTHY-5 | F |  |  | 1,7 | 18 |  | NO |
| HEALTHY-6 | M |  |  | 2,5 | 4,6 |  | NO |
| HEALTHY-7 | M |  |  | 8,9 | 31,9 |  | NO |
| HEALTHY-8 | F |  |  | 2,1 | 7,4 |  | NO |
| HEALTHY-9 | F |  |  | 3,8 | 6,9 |  | NO |
| HEALTHY-10 | M |  |  | 4,1 | 3,8 |  | NO |

* The HPS cases were categorized in terms of severity grades based on the following classification: Grade I for patients with prodromal symptoms without respiratory involvement; Grade II for patients with mild to moderate respiratory compromise without haemodynamic compromise; Grade III for patients with severe respiratory insufficiency with haemodynamic compromise; Grade IV for patients with severe respiratory insufficiency with refractory‐to‐treatment haemodynamic compromise, with a final fatal outcome.

** Renal dysfunction was assessed based on elevated serum creatinine levels exceeding 115 µmol/L
