# Supplementary material for: Hantavirus infection-induced B cell activation elevates free light chains levels in circulation
Source: PLoS Pathog. 2021 Aug 11;17(8):e1009843. doi: 10.1371/journal.ppat.1009843 (PMC8382192; doi:10.1371/journal.ppat.1009843)
Supplement: S3 Table — (DOCX) [file ppat.1009843.s009.docx]

S3 Table. Characteristics of patients with kidney biospies

| ID | Sex | Age | Diagnosis | Days after onset of fever | κ-LC expressing cells (%) | λ-LC expressing cells (%) |
| --- | --- | --- | --- | --- | --- | --- |
| HFRS-32 | F | 58 | ATIN | 11 | 4,4 | 4,6 |
| HFRS-33 | M | 45 | ATIN | 10 | 4,7 | 1,7 |
| HFRS-34 | F | 47 | ATIN | 7 | 4,6 | 2,4 |
| HFRS-35 | M | 38 | ATIN | 14 | 4,4 | 3,0 |
| HFRS-36 | M | 31 | no diagnosis | 16 | 2,2 | 0,5 |
| HFRS-37 | F | 50 | ATIN | 8 | 1,5 | 1,5 |
| HFRS-38 | M | 49 | ATIN | 15 | 6,7 | 4,8 |
| HFRS-39 | M | 38 | ATIN | 11 | 2,1 | 0,6 |
| HFRS-40 | F | 38 | ATIN | no info | 1,1 | 0,1 |
| HFRS-41 | M | 31 | ATIN | 12 | 1,6 | 3,1 |
| HFRS-42 | F | 33 | no diagnosis | 10 | 2,3 | 1,5 |
| HFRS-43 | M | 25 | ATIN | 12 | 5,2 | 4,9 |
| HFRS-44 | F | 62 | ATIN | 14 | 2,3 | 3,7 |
| HFRS-45 | F | 25 | no diagnosis | no info | 1,4 | 0,3 |
| HFRS-46 | M | 31 | AIN | 7 |  | 0,7 |
| HFRS-47 | M | 45 | AIN | no info | 3,2 | 2,3 |
| HFRS-48 | F | 34 | ATIN | 11 | 3,2 | 2,0 |
| HFRS-49 | M | 38 | no diagnosis | 17 | 1,5 | 0,3 |
| HFRS-50 | M | 27 | ATIN | no fever | 2,8 | 2,2 |
| HFRS-51 | M | 29 | ATIN | 13 | 1,8 | 0,7 |
| HFRS-52 | F | 39 | ATIN | 8 | 7,9 | 5,6 |
| HFRS-53 | M | 22 | ATIN | 11 | 3,5 | 1,6 |
| HFRS-54 | M | 20 | ATIN | 16 | 5,6 | 4,5 |
| HFRS-55 | M | 37 | ATIN | 9 | 2,7 | 1,3 |
| HFRS-56 | M | 30 | ATIN | 12 | 2,8 | 1,0 |
| HFRS-57 | M | 22 | ATIN | 12 |  | 1,1 |
| HFRS-58 | M | 25 | ATIN | 9 | 3,7 | 1,2 |
| HFRS-59 | M | 30 | AIN | 14 | 5,6 | 1,0 |
| HFRS-60 | F | 16 | ATIN | 12 | 3,5 | 1,7 |
| HFRS-61 | M | 27 | AIN | no info | 3,9 | 0,9 |
| HFRS-62 | F | 53 | ATIN | 13 |  | 4,5 |
| HFRS-63 | M | 40 | ATIN | 11 | 1,0 | 4,5 |
| HFRS-64 | M | 29 | ATIN | 10 | 8,4 | 5,2 |
| HFRS-66 | M | 30 | ATIN | 10 | 1,4 | 5,0 |
| HFRS-67 | M | 52 | ATIN | 13 | 3,5 | 5,0 |
| No HFRS-1 | F | 66 | IgAGN |  | 2,6 | 1,5 |
| No HFRS-2 | M | 73 | Mesangiocapill-GN |  | 2,4 | 0,6 |
| No HFRS-3 | M | 17 | ATIN |  | 1,4 | 0,6 |
| No HFRS-4 | M | 65 | Diab-NP |  | 5,4 | 0,2 |
| No HFRS-5 | F | 36 | MesProlif-GN |  | 2,8 | 1,5 |
| No HFRS-6 | M | 42 | Myeloma NP |  | 28,8 | 0,2 |
| No HFRS-7 | F | 18 | IgAGN |  | 1,2 | 0,2 |
| No HFRS-8 | M | 55 | ATIN |  | 11,6 | 0,2 |
| No HFRS-9 | F | 54 | Mesangiocapill-GN |  | 3,8 | 3,4 |
| No HFRS-10 | M | 28 | FSGS |  | 2,3 | 1,5 |
| No HFRS-11 | M | 72 | ATIN |  | 0,2 |  |
| No HFRS-13 | M | 34 | no diagnosis |  | 2,8 | 1,7 |
| No HFRS-14 | F | 30 | no diagnosis |  | 2,6 | 2,8 |
| No HFRS-15 | F | 38 | Arteriosclerosis |  | 1,2 | 1,1 |
| No HFRS-16 | M | 51 | IgAGN |  | 5,0 | 5,1 |
| No HFRS-17 | F | 24 | AGN |  | 0,9 | 0,7 |
| No HFRS-18 | M | 30 | MGN |  | 1,5 | 0,9 |
| No HFRS-19 | M | 36 | Diab-NP |  |  | 0,4 |
| No HFRS-20 | M | 37 | ATIN |  | 7,2 | 2,5 |
| No HFRS-21 | F | 30 | no diagnosis |  | 0,8 | 0,1 |
| No HFRS-22 | M | 31 | IgAGN |  | 3,0 | 1,5 |
| No HFRS-23 | F | 35 | IgAGN |  | 1,4 | 0,1 |
| No HFRS-24 | F | 76 | Mesangiocapill-GN |  | 4,0 | 1,4 |
| No HFRS-25 | F | 44 | no diagnosis |  | 1,2 | 0,1 |
| No HFRS-26 | F | 46 | AGN |  | 2,2 | 0,6 |
| No HFRS-27 | M | 74 | Diab-NP |  | 3,5 | 0,8 |
| No HFRS-28 | F | 47 | SLE-nephritis |  | 1,7 |  |
| No HFRS-29 | M | 42 | Arteriosclerosis |  | 3,9 | 0,2 |
| No HFRS-30 | M | 62 | Amyloidosis |  | 3,8 | 1,2 |
| No HFRS-31 | F | 20 | FSGS |  | 4,1 | 0,7 |
| No HFRS-32 | M | 42 | AGN |  | 5,4 | 0,7 |
| No HFRS-34 | F | 46 | Mesanigocapill-GN |  | 7,4 | 3,2 |
| No HFRS-35 | M | 59 | IgAGN |  | 3,1 | 0,6 |
| No HFRS-36 | M | 29 | no diagnosis |  | 2,1 | 0,3 |
| No HFRS-37 | M | 38 | IgAGN |  | 0,5 | 0,8 |
| No HFRS-38 | M | 41 | Diab-NP |  | 0,8 | 2,5 |
| No HFRS-39 | F | 30 | Diab-NP |  | 1,7 | 2,1 |
| No HFRS-41 | F | 43 | MesProlif-GN |  | 0,2 | 0,3 |
| No HFRS-42 | F | 39 | Diab-NP |  | 0,4 | 0,7 |
| No HFRS-43 | M | 18 | MGN |  | 0,1 | 0,3 |

AGN = Acute exudative glomerulonephritis

AIN = Acute interstitial nephritis

ATIN = Acute tubulointerstitial nephritis

Diab-NP = Diabetic nephropathy

FSGS = Focal segmental glomerulosclerosis

IgAGN = IgA glomerulonephritis

Mesangiocapill-GN = Mesangiocapillary glomerulonephritis

MesProlif-GN = Mesangial proliferative glomerulonephritis

MGN = Membranous glomerulonephritis

Myeloma-NP = Myeloma nephropathy

SLE-nephritis = Systemic lupus erythematosus nephritis
