# Supplementary material for: Hantavirus infection-induced B cell activation elevates free light chains levels in circulation
Source: PLoS Pathog. 2021 Aug 11;17(8):e1009843. doi: 10.1371/journal.ppat.1009843 (PMC8382192; doi:10.1371/journal.ppat.1009843)
Supplement: Attachment — Submitted filename: Responses to reviewers-TS-JH_sm.docx [file ppat.1009843.s010.docx]

**Re: PLoS Pathogens, Hantavirus Infection-Induced B Cell Activation Elevates Free Light Chains Levels in Circulation, PPATHOGENS-D-21-00682**

Dear Mr. Strandin,

Thank you very much for submitting your manuscript "Hantavirus Infection-Induced B Cell Activation Elevates Free Light Chains Levels in Circulation" for consideration at PLOS Pathogens. As with all papers reviewed by the journal, your manuscript was reviewed by members of the editorial board and by several independent reviewers. In light of the reviews (below this email), we would like to invite the resubmission of a significantly-revised version that takes into account the reviewers' comments.

We cannot make any decision about publication until we have seen the revised manuscript and your response to the reviewers' comments. Your revised manuscript is also likely to be sent to reviewers for further evaluation.

- *We thank the editorial board members and reviewers for providing the option to resubmit a revised version of our manuscript. As detailed below, we were able to address the concerns raised by reviewers with additional experiments and reformulating and amending the manuscript text. We hope that the editor(s) and reviewers share the opinion that the manuscript has improved after revisions, and that the revised version would meet the publications standards of PLoS Pathogens.*

**Reviewer #1:**

This manuscript builds on a recent publication by the same authors, where they reported PUUV-N protein binding FLCs in PUUV-infected patients. In this follow-up study Hepojoki and colleagues show a general increase of FLCs in blood of HFRS and HPS patients. During acute HFRS they observe increased FLC levels in blood and urine, and correlations between FLC levels with markers of kidney failure. Further, they show that PUUV infect B cells, which lead to secretion of FLCs. The findings are novel in a hantavirus perspective and add to previous reports of FLC secretion from activated B cells.

A strength of the study is the inclusion of HFRS and HPS patient samples, and the exploration of a potentially novel finding that can play a role in hantavirus pathogenesis.

A weakness of the study is that although the authors report a correlations between levels of FLCs and kidney damage, the potential role of FLCs in HFRS/HPS pathogenesis is unclear – does secreted FLCs represent more of a general response to immune activation (not specific for HFRS)? Moreover, an important finding is the potential infection of B cells. While the presented data do strongly suggest that B cells are infected, further in vitro analysis, with more specific methods, are needed to conclude this.

- *We thank the reviewer for the positive feedback. We agree with the reviewer in that we were unable to demonstrate a direct causality between upregulated FLCs and HFRS/HPS pathogenesis, this was indeed something we initially aimed for. Our hypothesis is that FLC upregulation would not be specific to HFRS or HPS, but might be something generally seen in (zoonotic?) virus infections. FLCs induce various pathological manifestations, and we like to speculate that the manifestation could relate to the target antigens of FLCs. We also find it possible that local activation (lungs in HPS and kidneys in HFRS) of B cells contributes to the associated manifestation.*

1.The finding of increased FLCs in circulation, and correlations to AKI, is interesting. However, to better understand potential mechanisms behind severity of HFRS/HPS, especially if FLCs play a role in pathogenesis, it would be interesting to compare levels of FLC in HPS-patients with fatal outcome vs survivors, and for HFRS-patients analyse for possible correlations to severity (for instance IL-6 levels and/or other markers of severity). Including these analysis would strengthen a possible role for FLC in pathogenesis/severity during hantavirus infection.

- *We thank the reviewer for the suggestions. We have compared the FLC levels to other clinical parameters measured from the same samples (for the hospitalized PUUV patients), but in the manuscript we only discussed the parameters that showed statistically significant correlation to FLC levels. However, we do agree with the reviewer the importance to report the association of FLC levels over a broad range of disease severity parameters and have now included a supplementary correlation matrix between FLC levels and various clinical and laboratory parameters reflecting disease severity (including IL-6, Supplementary Fig 2A). In addition, we compared FLC levels in HFRS patients with or without the need of* *dialysis treatment (Supplementary Fig. 3A).*

*We did also test the FLC kits for serum samples of infected macaques of earlier studies* [*https://pubmed.ncbi.nlm.nih.gov/11739712/*](https://pubmed.ncbi.nlm.nih.gov/11739712/) *and* [*https://pubmed.ncbi.nlm.nih.gov/18389907/*](https://pubmed.ncbi.nlm.nih.gov/18389907/)*. We did not include this data to the manuscript because only lambda FLC detection kit cross-reacted well enough, and the result suggested that there indeed is a correlation between disease severity and the amount of FLCs, the FLC level of the macaque that had to be euthanized due to disease severity peaked at the time of euthanasia.*

*In case of HPS samples, we stratified the patients by the extent of pulmonary and hemodynamic manifestations as well as fatality (grades 1-4) similarly as in a previously published study exploiting the same sample set (Garcia et al., 2017, Supplementary Fig. 3B) but did not observe specific differences between patient groups.*

*These data are now referred to in the results part of the text associated with Fig.2.*

2. Figure 2. In figures 2C and 2D mean is shown, however, there is a large fluctuation in the values between samples and between time points, hence outliers can have a strong effect. I suggest showing median or geometric mean instead of mean. Further, in figures 2C and 2D, or in the legend, include number of patients analysed at the different time-points.

- *We have replaced mean ± SEM with median ± interquartiles range in the revised Figures 2C and 2D (along with Fig.3 for consistency) following reviewer’s suggestions. Interestingly, the appearance of the data changes somewhat and association between FLC ratio and creatinine is not evident anymore, which in fact is also in line with correlation analysis (Supplementary Fig. 2A). The number of patients at different time points are depicted in Supplementary Fig.1, and this is also stated in the revised figure legend.*

Does the k/l FLC ratio correlates to creatinine levels in HFRS-patients?

- *We report the correlation between FLC ratio (k/l) and creatinine level both for the single time point at hospitalization (Supplementary Fig. 2A) and longitudinal data (see text associated with Fig. 2). In both cases, no significant correlation between FLC ratio (k/l) and serum creatinine levels were observed.*

Are there data available for creatinine in HPS-patients? A comparison also on HPS patient data could indicate if the same pattern is observed in HFRS and HPS, or if it is unique for HFRS.

- *Unfortunately, serum creatinine values are not available for HPS cases.*

Infection of B cells with PUUV. In figure 6A PUUV N pos cells are shown. However, the staining for PUUV N+ cells is not very distinct. Histogram showing PUUV N could be an alternative way to show this data.

- *We thank the reviewer for good suggestions for improving the data. We did the corresponding histograms (see reviewer Fig. 1 below). However, since the percentage of PUUN positive cells is relatively low for most B cell donors (< 5 %), we feel the pseudocolor plots are more distinctive way of presentation (by showing individual cells vs. cell counts) and therefore prefer to keep the original plots in Fig.6A.*

*Reviewer Figure 1. Histograms showing PUUN expression in mock, UV-PUUV and PUUV-infected naïve and memory B cells. PUUN+ B cells are gated.*

In figure 2C one PUUV N positive CD19+ cell is shown. Please include a figure with data on frequency of infected cells. In lines 123-125 it is stated that the enriched B cells were sorted to 100% purity. How was this confirmed? If possible, please provide as a supplementary figure flow data showing purity after B cell enrichment. My concern is that if very few infected cells were observed, they might represent other cell types than B cells.

- *We have now added a histogram (Supplementary Fig. 6A) showing that the B cell enrichment protocol results in over 90 % B cell purity, as judged by CD19 expression. In fact, StemCell Technologies report close to 99% purity for used the pan-B cell enrichment kit, when counting both CD19+ and CD19-CD43+ B cells.*

*For the immunofluorescence (IF) experiment in the previous version of the manuscript, we FACS-sorted B cells based on CD19 expression and concluded that these are by definition 100% pure B cells. However, we agree with the reviewer that showing one cell without co-staining with a B cell marker is not optimal. Thus, we have now replaced the previous IF assay with a new set of experiments using the enriched B cells from healthy donors and confirming that the infected cells represent B cell lineage by co-staining for kappa and lambda light chains (LC) (Fig. 6C). As one can appreciate from some of the IF images shown, there are also some cells without light chain positivity in the preparation (which are not necessarily B cells), and acting as negative controls for LC staining. However, based on this IF experiment, we decided not to report infection frequencies, since we feel the FACS experiment (Fig. 6A and B) is a more specific and robust way of doing this. FACS also allows to gate out dead cells and assess PUUN expression in different B cell subsets, which is not easy with IF. We hope this additional evidence is more convincing in showing that B cells are infected with hantavirus*.

While the presented data strongly suggest B cells are infected, clear evidence is lacking. Additional assays are needed to conclude that B cells are successfully infected. This can be achieved by analysing for instance progeny virus production and/or levels of viral RNA over time which could confirm that B cells can be infected by PUUV, and/or support replication.

- *For the revised manuscript, we have included a western blot of enriched B cells demonstrating PUUV N protein, as well as increase in the amount of FLCs, at 5 days post infection. No N protein was detected in cells infected with UV-inactivated virus preparation, confirming that the observed N protein is a product of active replication and not derived from the initial virus stock. We included also 3 day time point in our analysis, in which we did not observe any N protein, but since the amount of cells at that time point were significantly lower compared to the later time point, we decided not to include this in the figure. As the reviewer suggests, progeny virus production would also be an elegant way of assessing replication. However, we feel it is unlikely that a few positive cells in this assay setup would produce detectable amounts of infectious progeny viruses. Additionally, the fact the B cells are cultivated in suspension significantly hampers removing the RNA background resulting from inoculation (the number of cells used –grown in suspension- makes it very difficult to wash the cells enough times following inoculation .*

If possible, it would also be interesting to see if ANDV can infect B cells, and if ANDV infection has similar effects as PUUV or not on FLC secretion.

- *Unfortunately, we do not have the possibility to conduct experimental work with live ANDV, although we do agree with the reviewer that such experiments might indeed be interesting.*

Lines 65-66. It is rather well established that the route from lungs to kidneys is explained by systemic spread of hantaviruses: the main target of hantaviruses are endothelial cells lining capillaries, and hence the virus can spread to all organs in the body.

- *We have amended the sentence in the revised manuscript.*

Lines 104-105. Which k and l LC-specific antibodies were used?

- *The LC-specific immunohistochemistry is provided as a service by HUSLAB diagnostics laboratory. Both rabbit polyclonal antibodies are commercially available from Dako (A0191 and A0193). We have included this information in materials and methods of the revised manuscript.*

Lines 118-119. The link to Milteny can be omitted.

- *We have removed the link.*

Lines 124-125.

- *We are not sure why reviewer highlights this sentence: “For a subset of samples, the enriched B cells were further sorted to 100% purity by BD Influx cell cytometer (Biomedicum Flow Cytometry Core Facility, University of Helsinki). However, since we are not using the data obtained by this method anymore in the revised manuscript, we have removed this sentence.*

Line 149. From which company is the secondary Ab?

- *We have included the information to the revised manuscript.*

Line 162. What conditioned medium was used?

- *We thank the reviewer for spotting this. There was a mistake in the text and plain medium with 10 % FCS and antibiotics was actually used. We have revised the manuscript.*

Figure 1. It is difficult to see the bar for interquartile ranges for HPS-patients.

- *We have edited the figure to better show the interquartile ranges.*

Lines 222-223. This sentence seems a little bit misplaced, maybe move to the next section?

- *We thank the reviewer for spotting this, and we have omitted this sentence.*

Line 241. This sentence is rather speculative, please rephrase.

- *We have revised the sentence from “To evaluate the role of FLCs in the induction of AKI” to “To evaluate the renal clearance of FLCs during HFRS”,….*

Figure 3. Lines 244-247. Please provide a supplementary figure showing the correlations, they are not obvious when looking at figure 3A.

- *We have included the suggested supplementary figure 4 in the revised manuscript.*

Lines 325-326. This sentence is very speculative, the authors have not shown that PUUV infection lead to activation of B cells to become PBs.

- *We agree with the reviewer in that we do not have enough evidence to make strong claims, however, we wish to express such hypothesis. We have revised from “Taken together these results suggest that PUUV can infect and activate a subset of naïve B cells to become PBs.” to “ Taken together these results suggest that PUUV can infect various B cells subsets in vitro and in patients”.*

Figure 6C. The size bar should be explained in the figure legend

- *We have amended the figure legend accordingly.*

**Reviewer #2:**

This study demonstrates some interesting observations of the increased presence of FLCs during PUUV infections. The science in sound however the major limitation is that this is merely an observational study. The authors should include a comparison group with severe HFRS disease, for example patients diagnosed with HTNV or DOBV infections. Instead, they refer to PUUV-infections as HFRS, which they technically are; however due to the mild presentation have been most commonly referred to PUUV cases as NE and HFRS is reserved for the more severe Old World hantavirus infections. If the authors could demonstrate differences in non-hantavirus infections vs PUUV vs HTNV infections this manuscript would be far more informative.

- *We thank the reviewer for the overall positive feedback. We agree with the reviewer that the study builds on observations, however, at the same time we would like to point that the study includes experimental work to test hypotheses (e.g. direct activation of B cells by hantaviruses). We also agree with the reviewer that the manuscript would be strengthened by including serum samples from HTNV or DOBV patients; however, we did not have such samples available for this study. However, PUUV can also cause the more severe forms of HFRS, including severe acute kidney injury (strongly elevated serum creatinine and need for dialysis), and we feel our patient cohort do also represent the more severe HFRS. In the revised manuscript, we have cross-correlated serum FLCs together with a variety of parameters reflecting disease severity (Supplementary Fig 2A) and analyzed differences between patients with and without the need for dialysis (Supplementary Fig. 3A). In any case, in the revised Fig. 1, we now indicate that the study included samples specifically from PUUV-caused HFRS (PUUV-HFRS) to make a clear distinction to the generally more severe DOBV and HTNV infections.*

*The comparison to non-hantavirus infections is also a good idea, in fact we did include samples of “other febrile illness, OFI” to the initial analysis of FLC levels (Figure 1A-C). We think that the phenomenon is not unique to hantaviruses. However, we think that it would be too extensive to start screening different viruses one by one, and on the other hand, it would be hard to justify the selection of just some viruses.*

In this reviewers opinion a group of HTNV or DOBV -HFRS is required for more appropriate comparisons.

- *We appreciate the reviewer’s suggestion; however, we did not have such samples available for this study.*

**Reviewer #3:**
Overall, this is a well written manuscript, with a strong experimental method, clear results and figures, and well thought out discussion. The data presented here is building on previously published observation regarding free light chains (FLCs) as diagnostic analytes for Hantavirus infections, more specifically examining the infection-mediated production of FLCs and how their increase is related to pathology.

There are numerous strengths to this manuscript. The use of human clinical samples taken from acute PUUV infections is particularly valuable in this context, as is the sequential data taken during the course of the infection and subsequent recovery period. The extremely in-depth and comprehensive analysis of the roles of FLCs is also impressive, with multiple experimental techniques employed. The experimental question regarding the role of B cell activation and FRCs levels related to acute PUUV disease is very well answered.

One weakness is the broad capture of all acute PUUV cases (and ANDV cases as well for the HPS data) under one umbrella. I would have liked to have seen a more granular breakdown in some of the data analysis to examine whether variables (if the researchers have access) such as sex, age of patient, viral load, antibody responses etc. affected the parameters measured here. Even if the data presented here is independent of these factors, that should be stated somewhere.

As mentioned previously, some further breakdown within the HFRS patients based on other variables would increase the strength of some aspects of this manuscript (if data is readily available). This would especially relate to the data generated for figure 1, 2, 3 and possibly 4.

- *We thank the reviewer for the positive feedback and appreciate the suggestions. We have included a correlation matrix for the single time point FLC data (in Fig. 1) and renal plasma cell analysis (in Fig.4) including several different parameters available for these patients (Supplementary Fig 2A and B). In addition, FLC level analysis between patients stratified based on disease severity (need for dialysis for HFRS and level of pulmonary/hemodynamic compromise for HPS, Supplementary Fig. 3) were included. Regarding the sequential data in Fig. 2, we have also included an additional panel showing thrombocyte and leukocyte counts (Fig. 2E).*

For clarity, somewhere in the first results paragraph it should be stated that the HPS serum comes from ANDV-infected patients. This should also be in the figure legend.

- *We amended Fig.1 to specifically indicate that PUUV-caused HFRS and ANDV-caused HPS were analyzed.*

-In Figure 2, it would be useful (if possible) to indicate what or where humans “normal” values for the measured analytes are.

- *We appreciate the reviewer’s suggestion, and we have included the “normal” range to the figures.*

-How were serum creatinine levels determined? This should be in the materials and methods.

- *Plasma creatinine was determined by Cobas Integra (F. Hoffmann-La Roche Ltd., Basel, Switzerland).* *We have included this information to the materials and methods of the revised manuscript.*
